# Hypertension and Diabetes in Non-Pregnant Women of Reproductive Age in the United States

**DOI:** 10.5888/pcd16.190105

**Published:** 2019-10-24

**Authors:** Olumayowa Azeez, Aniket Kulkarni, Elena V. Kuklina, Shin Y. Kim, Shanna Cox

**Affiliations:** 1 Division of Reproductive Health, National Center for Chronic Disease Prevention and Health Promotion, Centers for Disease Control and Prevention, Atlanta, Georgia

## Abstract

**Introduction:**

Diagnosis and control of chronic conditions have implications for women’s health and are major contributing factors to maternal and infant morbidity and mortality. This study estimated the prevalence of hypertension and diabetes in non-pregnant women of reproductive age in the United States, the proportion who were unaware of their condition or whose condition was not controlled, and differences in the prevalence of these conditions by selected characteristics.

**Methods:**

We used data from the 2011–2016 National Health and Nutrition Examination Survey to estimate overall prevalence of hypertension and diabetes among women of reproductive age (aged 20–44 y), the proportion who were unaware of having hypertension or diabetes, and the proportion whose diabetes or hypertension was not controlled. We used logistic regression models to calculate adjusted prevalence ratios to assess differences by selected characteristics.

**Results:**

The estimated prevalence of hypertension was 9.3% overall. Among those with hypertension, 16.9% were unaware of their hypertension status and 40.7% had uncontrolled hypertension. Among women with diabetes, almost 30% had undiagnosed diabetes, and among those with diagnosed diabetes, the condition was not controlled in 51.5%.

**Conclusion:**

This analysis improves our understanding of the prevalence of hypertension and diabetes among women of reproductive age and may facilitate opportunities to improve awareness and control of these conditions, reduce disparities in women’s health, and improve birth outcomes.

**Summary**

**What is already known on this topic?**

Hypertension and diabetes have major implications for perinatal maternal morbidity and mortality. Women with chronic hypertension face substantially increased risk of life-threatening maternal outcomes, and diabetes during pregnancy increases the risk for unfavorable maternal and infant outcomes.

**What is added by this report?**

This analysis improves our understanding of the prevalence of hypertension and diabetes among women of reproductive age and may facilitate opportunities to improve awareness and control of these conditions, improve disparities in women’s health, and improve birth outcomes.

**What are the implications for public health practice?**

Adequately addressing these conditions provides an opportunity to reduce disparities in women’s health and ultimately improve birth outcomes.

## Introduction

Hypertension is the leading risk factor for heart disease and stroke, the first and fourth major causes of death in the United States, respectively (1). Hypertension is a major contributing factor to maternal and fetal morbidity, complicating up to 5% of the estimated 4 million pregnancies in the United States annually (2). Hypertension at a young age increases the risk of heart disease over the lifetime (3–5). Women with chronic hypertension and women with pregnancy-related hypertension have an increased risk of life-threatening maternal and infant outcomes, such as placental abruption, pulmonary edema, stroke, renal failure, preterm birth, intrauterine growth restriction, and death (2,6).

Diabetes is another one of the most common chronic diseases in the United States (7). Approximately 13% of adults (aged ≥20) have diabetes; 9.6% have physician-diagnosed diabetes and 3.0% have undiagnosed diabetes (4). For women of reproductive age, detection and management of pre-existing diabetes can help avoid the risks for poor maternal and infant outcomes (8). Among women with pre-existing diabetes, failure to properly control diabetes before conception and during the initial trimester of pregnancy increases risks for major birth defects and stillbirths. Failure to properly control diabetes during the last 2 trimesters can lead to large-for-gestational-age babies, which could pose risks for both mother and child.

Overall, the prevalence of chronic disease among women of reproductive age in the United States has increased, and improving women’s health during their reproductive years may improve the health of future generations (9). The objective of this study was to estimate the prevalence of hypertension and diabetes in non-pregnant women of reproductive age in the United States, the proportion of these women who were unaware of their condition or whose condition was not controlled, and differences in the prevalence of these conditions by selected characteristics.

## Methods

This study used questionnaire and examination data from the 2011–2016 National Health and Nutrition Examination Survey (NHANES) for hypertension and diabetes. NHANES is a nationally representative cross-sectional survey designed to assess the health and nutritional status of the US civilian, noninstitutionalized population. The survey became continuous in 1999, and data are released in 2-year cycles. Our study included data on non-pregnant women of reproductive age (aged 20–44) who provided an in-home medical history and underwent physical examination and laboratory testing at a mobile examination center. All participants undergo an in-home interview, during which NHANES collects self-reported data on demographic characteristics, socioeconomic characteristics, and health outcomes, including information about and medications for hypertension. A subset of participants from the in-home interviews undergo physical examinations at a mobile examination center, during which blood pressure data are collected. A subset of participants from the physical examination have laboratory testing, during which data on fasting plasma glucose (FPG) and hemoglobin A_1c_ (HbA_1c_) are collected.

Pregnancy status was self-reported during the in-home interview. Those who reported that they were not pregnant or did not know their pregnancy status were given a urine pregnancy test. If the urine test was negative, the participant was coded as not pregnant. If the urine test result was positive, the participant was coded as pregnant. We excluded from analysis women coded as pregnant.

The data were weighted to account for selection and nonresponse. Of the 29,902 participants who completed the in-home interview, 3,613 were non-pregnant women of reproductive age (weighted estimate, 50,018,292) and 192 were pregnant women of reproductive age. Of the 28,695 participants examined, 3,477 were non-pregnant women of reproductive age and 192 were pregnant women of reproductive age. Of the 3,477 non-pregnant women, blood pressure readings were missing for 173 participants and information on blood pressure medication was missing for 1 participant; these participants were excluded from the analysis of hypertension. The resulting sample size for estimating the prevalence of hypertension was 3,303 participants.

Of the 28,695 participants examined at the mobile examination center, NHANES obtained 9,759 fasting blood samples from participants aged 12 or older. Of these 9,759 participants, 8,512 had a positive, nonzero fasting subsample weight. Of those 8,512 participants, 1,462 were non-pregnant women of reproductive age and 65 were pregnant women of reproductive age. Of the 1,462 non-pregnant women, 1 participant was excluded from analysis because she did not know if she was told she had diabetes. The resulting sample size for estimating diabetes was 1,461 (weighted estimate, 50,406,331). 

### Covariates

Demographic information collected during the in-home interview included age (categorized into 20–34, 35–39, or 40–44 y), self-identified race/ethnicity (non-Hispanic white, non-Hispanic black, Hispanic, or other), education level (<high school diploma, high school diploma, or >high school diploma), and health insurance (public, private, insurance, or uninsured). Body mass index (BMI) was calculated by using data on height and weight collected during physical examination (normal or underweight [BMI <25.0 kg/m^2^]; overweight [BMI from 25.0 to <30.0 kg/m^2^]; obese [BMI ≥30.0 kg/m^2^]).

### Hypertension

Hypertension was defined as having an average of up to 3 consecutive measurements of systolic blood pressure ≥140 mm Hg or diastolic blood pressure ≥90 mm Hg, or when participants reported currently using medication to lower blood pressure (10,11). Participants were asked during the in-home interview if they had ever been told by a doctor or other health professional that they had hypertension. Among participants who were identified as having hypertension, those that were never told of their hypertension status by a doctor or a health professional and were not taking prescribed blood pressure medicine were categorized as being unaware of their hypertension status. Uncontrolled hypertension was defined as systolic blood pressure ≥140 mm Hg and diastolic blood pressure ≥90 mm Hg among those with hypertension (11).

### Diabetes

In this study, we defined diabetes by using 2 laboratory values, FPG and HbA_1c_, and a self-report of diagnosed diabetes. We restricted the analytic sample to women who provided a fasting blood sample in the morning session of the mobile examination and had fasted for 8 to 23 hours. FPG values for 2011–2016 NHANES were calibrated to FPG values for 1999–2004 NHANES for comparability purposes; calibration was necessary because NHANES changed its methods for measuring FPG in 2005–2006. We considered participants to have diagnosed diabetes if they self-reported that they were told by a doctor or other health professional that they had diabetes. Participants were considered not to have diagnosed diabetes if they never received a diagnosis and had an HbA_1c_ ≥6.5% or an FPG ≥126 mg/dL. Uncontrolled diabetes was defined as having diagnosed diabetes and an HbA_1c_ ≥7% (12,13).

### Statistical analysis

We performed all analyses with SAS-callable SUDAAN version 9.3 (SAS Institute Inc) using appropriate sample weights, stratification, and clustering to account for the complex survey design. We calculated the distribution of participant characteristics and estimated the prevalence of hypertension, lack of awareness of hypertension status, uncontrolled hypertension, diabetes, undiagnosed diabetes, and uncontrolled diabetes, and 95% confidence intervals (CIs). Next, we estimated the prevalence of these conditions by participant characteristics (age group, race/ethnicity, education level, BMI, and insurance status). We used the χ^2^ test to assess homogeneity of distribution across each participant characteristic. Finally, we used PROC RLOGIST to calculate multivariable predicted marginal proportions from logistic regression models. We adjusted the resulting prevalence ratios (PRs) for all other covariates in the multivariable model. The covariates used in the models included all participant characteristics. We did not use BMI as a covariate in the model for uncontrolled diabetes because the value was zero for the category normal or underweight. Significance was determined by using a *P* value <.05.

## Results

The overall prevalence of hypertension among women of reproductive age was 9.3%; of these women, 16.9% were unaware of their hypertension status and 40.7% had uncontrolled hypertension (Table 1). The proportion of women with hypertension who reported using blood pressure medication was 70.0% (95% CI, 64.1%–75.4%). The prevalence of hypertension was higher among women aged 40 to 44 (adjusted PR = 5.1; 95% CI, 3.7–7.1) and women aged 35 to 39 (adjusted PR = 3.9; 95% CI, 3.0–5.2) than among women aged 20 to 34 and higher among non-Hispanic black women (adjusted PR = 1.7; 95% CI, 1.4–2.2) than among non-Hispanic white women. Hypertension was more common among obese women (adjusted PR = 3.8; 95% CI, 2.8–5.3) and overweight women (adjusted PR = 1.7; 95% CI, 1.1–2.5) than among normal-weight or underweight women (Table 2).

**Table 1:** Characteristics of Non-Pregnant Women Aged 20–44, NHANES 2011–2016

Characteristic	Weighted Denominator^a^	Weighted No.^b^	%^c^ (95% CI)
**Age, y**			
20–34	50,018,292	29,695,171	59.4 (56.6–62.1)
35–39		9,744,417	19.5 (17.9–21.2)
40–44		10,578,705	21.2 (19.2–23.3)
**Race/ethnicity**			
Non-Hispanic white	50,018,292	28,103,804	56.2 (51.2–61.1)
Non-Hispanic black		6,857,756	13.7 (11.0–17.0)
Hispanic		9,867,791	19.7 (16.4–23.6)
Other		5,188,941	10.4 (9.1–11.9)
**Education level **			
<High school diploma	50,018,292	6,266,820	12.5 (10.7–14.6)
High school diploma		8,493,558	17.0 (15.1–19.1)
>High school diploma		35,226,226	70.5 (67.0–73.7)
**Body mass index, kg/m^2^**			
Normal or underweight	49,906,433	18,885,653	38.2 (35.4–41.0)
Overweight		12,476,053	25.2 (23.4–27.1)
Obese		18,094,169	36.6 (34.8–38.4)
**Insurance status**			
Publicly insured	49,906,433	9,190,888	18.6 (16.4–20.9)
Privately insured		29,057,469	58.6 (55.5–61.7)
Uninsured		11,304,289	22.8 (20.6–25.2)
**Hypertension **			
Has hypertension	47,556,182	4,428,498	9.3 (8.1–10.7)
Is unaware of hypertension status	4,428,498	750,284	16.9 (13.2–21.5)
Has uncontrolled hypertension	4,428,498	1,803,248	40.7 (35.0–46.7)
**Diabetes**			
Has diabetes	50,376,524	2,256,582	4.5 (3.4–5.9)
Has diagnosed diabetes	50,376,524	1,606,530	3.2 (2.3–4.5)
Has uncontrolled diabetes	1,606,530	827,973	51.5 (37.0–65.8)
Has undiagnosed diabetes	50,376,524	650,052	1.3 (0.8–2.0)

Abbreviations: CI, confidence interval; NHANES, National Health and Nutrition Examination Survey.

a Values used for denominator in calculating percentages.

b Weighted numbers may not add up to the total denominator because of missing data or rounding.

c Percentages may not sum to 100% because of rounding.

**Table 2:** Prevalence and Adjusted Prevalence Ratios^a^ of Hypertension, Unawareness of Hypertension, and Uncontrolled Hypertension, by Selected Characteristics of Non-Pregnant Women Aged 20–44, NHANES, 2011–2016

Characteristic	Has Hypertension, % (Weighted n)	*P* b	Adjusted PR (95% CI)	Is Unaware of Hypertension, % (Weighted n)	*P* b	Adjusted PR (95% CI)	Has Uncontrolled Hypertension, % (Weighted n)	*P* b	Adjusted PR (95% CI)
**Age, y**									
20–34	3.6 (998,940)	<.001	1.0 [Ref]	26.0 (259,428)	.20	1.0 [Ref]	55.7 (556,662)	.06	1.0 [Ref]
35–39	15.1 (1,412,725)		3.9 (3.0–5.2)	15.9 (223,874)		0.7 (0.4–1.2)	37.7 (532,118)		0.8 (0.5–1.1)
40–44	20.0 (2,016,833)		5.1 (3.7–7.1)	13.2 (266,982)		0.5 (0.2–0.9)	35.4 (714,468)		0.7 (0.5–0.9)
**Race/ethnicity**									
Non-Hispanic white	8.2 (2,208,478)	<.001	1.0 [Ref]	16.4 (363,102)	.17	1.0 [Ref]	29.6 (654,144)	<.001	1.0 [Ref]
Non-Hispanic black	18.7 (1,225,450)		1.7 (1.4–2.2)	14.9 (182,530)		1.0 [Ref] (0.5–2.1)	53.6 (657,246)		1.7 (1.2–2.4)
Hispanic	7.5 (690,525)		0.8 (0.6–1.0)	14.5 (100,000)		1.0 (0.5–2.2)	44.3 (305,668)		1.3 (0.8–2.1)
Other	6.2 (304,045)		0.9 (0.7–1.4)	34.4 (104,652)		2.1 (1.0–4.3)	61.2 (186,190)		2.0 (1.3–3.0)
**Education level**									
<High school diploma	12.0 (705,717)	.03	1.0 [Ref]	15.5 (109,438)	.91	1.0 [Ref]	42.2 (297,802)	.93	1.0 [Ref]
High school diploma	11.3 (905,371)		1.0 (0.7–1.5)	19.0 (171,687)		1.3 (0.6–2.9)	42.8 (387,276)		1.1 (0.7–1.6)
>High school diploma	8.4 (2,817,410)		0.8 (0.6–1.1)	16.7 (469,159)		0.9 (0.4–1.9)	39.7 (1,118,171)		1.0 (0.6–1.5)
**Body mass index**									
Normal or underweight	3.4 (622,142)	<.001	1.0 [Ref]	39.9 (247,918)	.10	1.0 [Ref]	60.3 (374,904)	.02	1.0 [Ref]
Overweight	6.5 (776,485)		1.7 (1.1–2.5)	10.3 (80,160)		0.3 (0.1–0.6)	23.2 (18,0069)		0.4 (0.2–0.7)
Obese	17.2 (2,981,356)		3.8 (2.8–5.3)	14.2 (422,206)		0.4 (0.2–0.8)	41.4 (1,235,550)		0.6 (0.5–0.9)
**Insurance status**									
Privately insured	9.2 (2,552,408)	.07	1.0 [Ref]	17.4 (443,592)	.02	1.0 [Ref]	36.4 (929,885)	.003	1.0 [Ref]
Publicly insured	12.0 (1,039,649)		1.1 (0.9–1.4)	8.9 (92,319)		0.5 (0.2–0.8)	31.4 (326,534)		0.7 (0.4–1.0)
Uninsured	7.4 (797,966)		0.8 (0.5–1.3)	25.2 (200,902)		1.2 (0.7–2.2)	63.7 (508,354)		1.5 (1.1–2.1)

Abbreviations: CI, confidence interval; NHANES, National Health and Nutrition Examination Survey; PR, prevalence ratio; Ref, reference.

a Adjusted for all other variables in analysis.

b Determined by χ^2^ test; *P* < .05 considered significant.

The prevalence of being unaware of hypertension status was lower among women aged 40 to 44 (adjusted PR = 0.5; 95% CI, 0.2–0.9) than among women aged 20 to 34, among obese women (adjusted PR = 0.4; 95% CI, 0.2–0.8) and overweight women (adjusted PR = 0.3; 95% CI, 0.1–0.6) than among normal-weight or underweight women, and among publicly insured women (adjusted PR = 0.5; 95% CI, 0.2–0.8) than among privately insured women (Table 2). The prevalence of uncontrolled hypertension was lower among women aged 40 to 44 (adjusted PR = 0.7; 95% CI, 0.5–0.9) than among women aged 20 to 34 and higher among women of “other” race/ethnicity (adjusted PR = 2.0; 95% CI, 1.3–3.0) and non-Hispanic black women (adjusted PR = 1.7; 95% CI, 1.2–2.4) than among non-Hispanic white women. The prevalence of uncontrolled hypertension was also lower among obese women (adjusted PR = 0.6; 95% CI, 0.5–0.9) and overweight women (adjusted PR = 0.4; 95% CI, 0.2–0.7) than among normal-weight or underweight women and was higher among uninsured women (adjusted PR = 1.5; 95% CI, 1.1–2.1) than among privately insured women (Table 2).

The overall prevalence of diabetes among non-pregnant women of reproductive age was 4.5%; the prevalence of diagnosed diabetes was 3.2%, and the prevalence of undiagnosed diabetes was 1.3%. Thus, approximately 30% of women with diabetes had undiagnosed diabetes. Among women with diagnosed diabetes, the prevalence of uncontrolled diabetes was 51.5% (Table 1). Diabetes was more common among women aged 40 to 44 (adjusted PR = 3.1; 95% CI, 1.7–5.8) and women aged 35 to 39 (adjusted PR = 2.2; 95% CI, 1.2–4.1) than among women aged 20 to 34, among women of “other” race/ethnicity (adjusted PR = 2.6; 95% CI, 1.1–6.0) and non-Hispanic black women (adjusted PR = 2.0; 95% CI, 1.1–3.5) than among non-Hispanic white women, and among obese women (adjusted PR = 13.5; 95% CI, 4.4–41.5) and overweight women (adjusted PR = 5.5; 95% CI, 1.6–18.8) than among normal-weight or underweight women. Women of “other” race/ethnicity (adjusted PR = 8.6; 95% CI, 2.1–35.2), non-Hispanic black women (adjusted PR = 6.1; 95% CI, 2.0–18.6), and Hispanic women (adjusted PR = 3.7; 95% CI, 1.2–11.1) had a higher prevalence of undiagnosed diabetes than did non-Hispanic white women, as did obese women (adjusted PR = 7.2; 95% CI, 1.7–30.2) compared with normal-weight or underweight women (Table 3).

**Table 3:** Prevalence and Adjusted Prevalence Ratios^a^ of Diabetes, Undiagnosed Diabetes, and Uncontrolled Diabetes, by Selected Characteristics of Non-Pregnant Women Aged 20–44, NHANES, 2011–2016

Characteristic	Has Diabetes, % (Weighted n)	*P* b	Adjusted PR (95% CI)	Has Undiagnosed Diabetes, % (Weighted n)	*P* b	Adjusted PR (95% CI)	Has Uncontrolled Diabetes, % (Weighted n)	*P* b	Adjusted PR (95% CI)
**Age, y**									
20–34	2.5 (738,995)	<.001	1.0 [Ref]	0.9 (253,807)	<.001	1.0 [Ref]	44.8 (217,548)	.76	1.0 [Ref]
35–39	6.0 (593,406)		2.2 (1.2–4.1)	1.9 (185,957)		2.0 (0.8–5.1)	61.6 (250,819)		1.9 (0.7–4.9)
40–44	8.7 (924,181)		3.1 (1.7–5.8)	2.0 (210,288)		2.2 (0.8–5.7)	50.4 (359,606)		1.8 (0.7–4.4)
**Race/ethnicity**									
Non-Hispanic white	3.0 (860,012)	.009	1.0 [Ref]	0.4 (123,066)	<.001	1.0 [Ref]	54.0 (398,251)	.047	1.0 [Ref]
Non-Hispanic black	8.2 (562,833)		2.0 (1.1–3.5)	3.1 (211,662)		6.1 (2.0–18.6)	72.5 (254,676)		1.4 (0.9–2.2)
Hispanic	5.9 (575,823)		1.5 (0.8- 2.7)	2.0 (198,901)		3.7 (1.2–11.1)	42.5 (160,056)		0.9 (0.3–2.3)
Other	5.1 (257,914)		2.6 (1.1–6.0)	2.3 (116,424)		8.6 (2.1–35.2)	10.6 (14,990)		0.2 (0.02–1.1)
**Education level**									
<High school diploma	5.9 (422,084)	.48	1.0 [Ref]	2.4 (173,923)	.004	1.0 [Ref]0	30.5 (75,688)	.48	1.0 [Ref]
High school diploma	5.3 (427,406)		1.1 (0.5–2.5)	1.1 (90,602)		0.5 (0.1–2.1)	61.4 (206,618)		1.6 (0.6–4.3)
>High school diploma	4.0 (1,407,092)		1.0 (0.5–1.9)	1.1 (385,528)		0.7 (0.3–1.7)	53.4 (545,667)		1.3 (0.5–3.5)
**Body mass index**									
Normal or under weight	0.6 (108,414)	<.001	1.0 [Ref]	0.3 (59,281)	<.001	1.0 [Ref]	0 (0)	.32	—c
Overweight	3.5 (432,412)		5.5 (1.6–18.8)	0.8 (99,323)		2.2 (0.3–15.5)	63.7 (211,997)		—c
Obese	8.9 (1,688,792)		13.5 (4.4–41.5)	2.5 (478,503)		7.2 (1.7–30.2)	50.9 (615,976)		—c
**Insurance status**									
Privately Insured	3.7 (1,041,739)	.14	1.0 [Ref]	0.9 (259,382)	.002	1.0 [Ref]	61.4 (480,365)	.47	1.0 [Ref]
Publicly Insured	5.6 (543,488)		1.2 (0.6–2.4)	1.6 (151,835)		1.0 (0.4–2.7)	40.4 (158,285)		0.5 (0.2–1.1)
Uninsured	5.6 (656,946)		1.4 (0.8–2.2)	2.0 (238,835)		1.5 (0.6–3.7)	41.8 (174,914)		0.6 (0.3–1.2)

Abbreviations: CI, confidence interval; NHANES, National Health and Nutrition Examination Survey; PR, prevalence ratio; Ref, reference.

a Adjusted for all other variables in analysis.

b Determined by χ^2^ test; *P* < .05 considered significant.

c Body mass index not used as a covariate in the model for uncontrolled diabetes because of insufficient sample size.

## Discussion

Our analysis used NHANES data to provide prevalence estimates on 2 major chronic conditions relevant to preconception and overall health among women. In this representative sample of non-pregnant women of reproductive age in the United States, the overall prevalence of hypertension was 9.3% and the overall prevalence of diabetes was 4.5%. Approximately 17% of women with hypertension in our sample were unaware that they had it, and more than 40% had uncontrolled hypertension. We also found that almost 30% of women with diabetes never received a diagnosis, and among women with diagnosed diabetes, more than half had uncontrolled diabetes.

The high proportion of undiagnosed diabetes may be due partially to an absence of well-defined screening recommendations for diabetes among women of reproductive age. The US Preventive Services Task Force and the American Diabetes Association recommend screening adults aged 45 or older who are overweight or obese (13,14). These guidelines mention that people with certain risk factors (ie, family history of diabetes, history of gestational diabetes or polycystic ovarian syndrome, or members of certain racial/ethnic groups) may be at increased risk for diabetes at a younger age. However, recommendations for screening among younger patients with one or more risk factors for diabetes remains at the discretion of health care providers (13). The American Diabetes Association also provides guidelines for testing children and adolescents for prediabetes and type 2 diabetes: children and adolescents aged 18 or younger who are overweight or obese and have at least 1 additional risk factor should be considered (14). However, no guidelines specify the age range (20–44 y) assessed in our analysis.

In contrast to recommendations for diabetes, the US Preventive Services Task Force recommends screening all adults (≥18 y) for hypertension (13). Although the prevalence of hypertension in our study was higher among women aged 40 to 44 (than among women aged 20 to 34) and among overweight or obese women (than among normal-weight or underweight women), these women were less likely to be unaware of their hypertension status or have uncontrolled hypertension. Older age and overweight/obesity are well-established risk factors for hypertension and other chronic conditions (15). Thus, older women and women with high BMIs may have more health care encounters and more opportunities for hypertension screening and therefore have better access to care for hypertension management.

The prevalence of hypertension and diabetes increased with age and BMI in our study sample, similar to increases among the general population (13). Although we found no difference in the prevalence of hypertension by insurance status, publicly insured women were less likely than privately insured women age to be unaware of their hypertension status and uninsured women were more likely to have uncontrolled hypertension. Women who lack health insurance may have less access to care and face more challenges in obtaining resources to manage chronic health conditions. We also found that non-Hispanic black women were more likely than non-Hispanic white women to have both hypertension and uncontrolled hypertension and that diabetes prevalence was higher among non-Hispanic black women and women of “other” race/ethnicity than among non-Hispanic white women. Among the 4 racial/ethnic groups studied, the prevalence of undiagnosed diabetes was lowest among non-Hispanic white women.

Our results demonstrated that a substantial proportion of women of reproductive age with hypertension and diabetes do not have these conditions under control (hypertension, 40.7% and diabetes, 51.5%). Although hypertension and diabetes are often considered a public health problem among older adults, these conditions can also have long-term consequences for younger adults. Adults who maintain or decrease their blood pressure to normal levels by and during middle age (41–55 y) have the lowest lifetime risk for cardiovascular disease (ie, fatal coronary heart disease, hospitalized myocardial infarction, nonhospitalized myocardial infarction, and fatal and nonfatal stroke [16]), compared with adults who do not maintain or decrease their blood pressure to normal levels.

Length of time living with diabetes is linearly associated with increased risk of fatal coronary heart disease among women aged 30 to 55 (17). Any chronic hypertension, regardless of severity and level of control, is associated with adverse maternal and fetal outcomes (18), including pre-eclampsia and preterm birth. However, women with uncontrolled blood pressure have a greater risk of adverse maternal and fetal outcomes than women with controlled hypertension at less than 20 weeks gestational age (19). Pre-existing maternal diabetes (type 1 and type 2) is associated with an increased risk of congenital malformations during pregnancy, and up to the seventh gestational week, with HbA_1c_ in the range of 6.3% to 11% (45–97 mmol/mol); pre-existing maternal diabetes is the most important predictor of congenital malformations (20). Pre-existing diabetes is also associated with an increased risk of adverse outcomes, including stillbirth, macrosomia (birthweight >4,000 g), caesarean section, and shoulder dystocia (21). Additionally, gestational diabetes increases the risk for future type 2 diabetes (22).

Ideally, diagnosing and achieving optimum control of chronic conditions, including diabetes and hypertension, among women of reproductive age should be a part of preconception care (23). For women who want to delay or avoid becoming pregnant, the 2016 US Medical Eligibility Criteria for Contraceptive Use has recommendations on contraception for men and women with certain medical conditions, including hypertension and diabetes (24). Additionally, providers can screen for and treat complications as well as modify medications for their potential teratogenic effects (20).

Health care providers and public health professionals can improve diagnosis and control of hypertension and diabetes among women of reproductive age by providing health education, particularly to women at risk for these conditions, including overweight and obese women and racial/ethnic minorities. They also can work with women with hypertension and diabetes after diagnosis to properly manage their conditions. Evidence-based programs such as the National Diabetes Prevention Program and the Million Hearts initiative address diabetes and hypertension; however, these programs are geared toward an older population. Connecting women to interventions or programs that are targeted to women of reproductive age, such as The Magnolia Project, can facilitate health education and screening services for these women, especially women who lack access to a regular source of care (25). Future interventions should seek to overcome barriers that are specific to racial/ethnic minority groups and incorporate culturally appropriate, respectful care and services that address the social determinants of health (eg, transportation, housing insecurity) (26).

Our study has several limitations. Because we restricted our diabetes analysis to women who provided a fasting blood sample, we had a small sample size, which resulted in wide confidence intervals for some comparisons and limited our ability to detect true differences. In addition, we combined multiple years of data to achieve sufficient sample size for the analysis overall, limiting the ability to disentangle the effect of trends (3–5,8) of increasing prevalence of these conditions among women of reproductive age. Some covariates of interest, such as insurance status, may be transient and change over time.

An additional limitation is that we examined hypertension and diabetes in non-pregnant women; we did not assess history of pregnancy-induced hypertension or diabetes, which are associated with development of these chronic conditions later in life. Future analyses could explore these areas. Overall, however, our analysis provides advantages over other analyses of women of reproductive age because it used both self-reported and standardized measured data.

Our study was based on recent data and improves our understanding of disparities in the prevalence, diagnosis, and control of hypertension and diabetes among women of reproductive age. These disparities mirror disparities in maternal and infant outcomes. Findings from our study may facilitate opportunities to improve awareness and control of these conditions among women of reproductive age and improve the long-term health outcomes of women later in life. Adequately addressing these conditions may also potentially improve inequities in birth outcomes.
